# Deficiency in Silicon Transporter Lsi1 Compromises Inducibility of Anti-herbivore Defense in Rice Plants

**DOI:** 10.3389/fpls.2019.00652

**Published:** 2019-05-24

**Authors:** Yibin Lin, Zhongxiang Sun, Zhenfang Li, Rongrong Xue, Weikang Cui, Shaozhi Sun, Tingting Liu, Rensen Zeng, Yuanyuan Song

**Affiliations:** ^1^Key Laboratory of Ministry of Education for Genetics, Breeding and Multiple Utilization of Crops, College of Crop Science, Fujian Agriculture and Forestry University, Fuzhou, China; ^2^Institute of Crop Resistance and Chemical Ecology, College of Life Sciences, Fujian Agriculture and Forestry University, Fuzhou, China

**Keywords:** silicon, *Oryza sativa*, rice leaffolder, induced defense, silicon-transporter *OsLsi1*, jasmonate signaling

## Abstract

Silicon (Si) application can significantly enhance rice resistance against herbivorous insects. However, the underlying mechanism is elusive. In this study, silicon transporter mutant *OsLsi1* and corresponding wild-type rice (WT) were treated with and without Si to determine Si effects on rice resistance to leaffolder (LF), *Cnaphalocrocis medinalis* (Guenée) (Lepidoptera: Pyralidae). Si application on WT plants significantly promoted rice plant growth, upregulated expression level of *OsLsi1* and increased Si accumulation in the leaves and roots, as well as effectively reduced LF weight gain, while it showed only marginal or no effect on the mutant plants. Furthermore, upon LF infestation, transcript levels of *OsLOX*, *OsAOS2*, *OsCOI1a*, *OsCOI1b*, and *OsBBPI*, and activity of catalase, superoxide dismutase, peroxidase, and polyphenol oxidase were significantly higher in Si-treated than untreated WT plants. However, *OsLsi1* mutant plants displayed higher susceptibility to LF, and minimal response of defense-related enzymes and jasmonate dependent genes to Si application. These results suggest that induced defense plays a vital role in Si-enhanced resistance and deficiency in silicon transporter Lsi1 compromises inducibility of anti-herbivore defense in rice plants.

## Introduction

In the last century rapid industrialization and urbanization have led to dramatic ecological and biochemical changes in the environment, which have various detrimental effects on quality and productivity of agricultural crops (Godfray et al., 2010). This situation is further worsened by the increasing food demands from the ever-increasing population (Liang et al., 2015). Insect herbivory is one of the major problems in crop production worldwide, therefore appropriate management of insect pests is of particular importance for sustainable agriculture (Sanchis and Bourguet, 2008). For more than half a century the use of pesticides has been considered the most effective way to manage insect pests in crop production (Glare et al., 2012). However, the abuse of pesticides has also brought an array of problems such as environmental pollution (Rockstrom et al., 2009), pest resistance (Wu and Guo, 2005), food safety (Glare et al., 2012), etc. Alternative strategies are urgently required to maintain agricultural sustainability (Glare et al., 2012).

Silicon (Si) is abundant in nature and second only to oxygen in the crust (28.8%) (Sommer et al., 2006). Although Si is not considered an essential element of plants, the ability of Si to significantly enhance crop resistance to both biotic and abiotic stresses as a “beneficial element” has been widely demonstrated (Ma et al., 2006; Guntzer et al., 2012; Meharg and Meharg, 2015). Although silicate minerals are dominant soil components, the bioavailable silicic acid is scarce particularly in croplands due to continuous Si remove with crop harvest (Meena et al., 2014). In addition, strong weathering also leads to scarcity of Si in soils of tropical and subtropical regions, which are the main rice producing regions (Savant, 1997). Therefore, application of Si fertilizer in agriculture is gaining attention. Si-mediated alleviation of abiotic stresses such as nutrient imbalance (Ma and Takahashi, 1991), salinity (Rios et al., 2017), drought (Devrim et al., 2016) and heavy metals (Chen et al., 2019) has been extensively reported. Besides, Si has also been widely reported to significantly enhance crop resistance against diseases and pests (Reynolds et al., 2016; Wang et al., 2017).

As early as the 1950s, it was reported that Si enhanced plant resistance to insect pests (Sasamoto, 1958; Liang et al., 2015). Since then, an array of studies have shown that Si can enhance anti-herbivore resistance of many important crops (Liang et al., 2015). Physical barrier has been considered as a main mechanism of Si-enhanced anti-herbivore resistance (Salim and Saxena, 1992; Massey and Hartley, 2009). The silicon absorbed by plant roots is transported to the plant shoots, and deposited in the veins and leaf epidermis to form dense single or double rows of silicon chains, which thickens the cell wall and increases roughness of leaves, thus forming a physical barrier (Liang et al., 2015). Silica accumulation increases abrasiveness of plant tissues and thereby deters feeding, as well as reduces foliage digestibility and herbivore performance (Massey and Hartley, 2009; Keeping et al., 2014). It also affects insect digestion efficiency and feeding behavior by wearing mouthparts (Massey et al., 2006).

Recent studies show that in addition to physical defense, Si enhances plant resistance to insects by inducing biochemical and molecular defense responses (Ye et al., 2013). By using microarray Fauteux et al. (2006) found that in powdery mildew-challenged *Arabidopsis* plants Si induced defense responses of various defense-related genes included R genes, stress-related transcription factors, genes involved in signal transduction, the biosynthesis of stress hormones (SA, JA, ethylene), and the metabolism of reactive oxygen species. In *Ralstonia solanacearum* inoculated tomato plants Si amendment showed similar induced effects (Ghareeb et al., 2011). Furthermore, Si application enhances natural enemy attraction to pest-infested plants and thus improves biological control (Kvedaras et al., 2010; Liu et al., 2017). Si enhances rice resistance to the brown spot fungus *Cochliobolus miyabeanus* by preventing the pathogen from hijacking the plant ethylene pathway (Van Bockhaven et al., 2015). Si also delays leaf senescence in *Arabidopsis* and *Sorghum* via increased cytokinin biosynthesis (Markovich et al., 2017).

All terrestrial plants can accumulate a certain amount of Si, but its concentrations vary greatly with species (Richmond and Sussman, 2003). In rice plants Si accumulation can reach up to 10% of dry weight, which is much higher than the accumulation of other mineral nutrients (Ma and Takahashi, 2002). The uptake of most Si by rice roots is an active process mediated by two types of silicon transporters, influx and efflux. The transporter Lsi (Low silicon rice) is polarly distributed in rice root tissue. Lsi1 localizes to the lateral plasma membrane of both exodermis cells and endodermis cells (Casparian band), and it is responsible for transporting orthosilicic acid [Si(OH)_4_] in the external solution into cortical cells (Yamaji and Ma, 2007). Lsi2 is located in the medial plasma membrane of Casparian band cells, which is responsible for transporting Si into the apoplast of the aerenchyma (Yamaji and Ma, 2011). The synergy of Lsi1 and Lsi2 in the endodermis transports Si into the root stele. The Si in the xylem vessel is transported to the shoot by the transpiration flow, and then by Lsi6, which is located in the xylem parenchyma cells of the sheath and leaves, is responsible for unloading and dispensing the Si in the xylem (Yamaji and Ma, 2008).

The jasmonate (JA) signaling pathway plays a vital role in mediating plant defense responses to insect herbivory (Howe and Jander, 2008). Our previous study demonstrates that there exists strong interaction between Si and JA in rice defense responses to caterpillar *Cnaphalocrocis medinalis* (rice leaffolder, LF) infestation (Ye et al., 2013). We hypothesized that low Si accumulation will impair inducibility of anti-herbivore defense in rice plants. In this study, Si transporter deficient mutant *OsLsi1* and the corresponding wild type (WT) were used to compare their defense responses to LF infestation in rice plants with or without Si amendment. Upon LF attack, Si-treated WT plants exhibited increased defense responses relative to untreated controls, including elevated levels of transcripts encoding marker genes of JA pathway; and increased activities of catalase, superoxide dismutase, peroxidase, and polyphenol oxidase. On the other hand, the Si-treated mutant *OsLsi1* plants exhibited only marginal or no induction in response to LF infestation. Additionally, significant reductions in Si deposition and an apparent loss of Si-mediated LF resistance were observed in *OsLsi1* mutant plants. Our results demonstrate that Si can enhance rice defense against chewing insect LF and deficiency in silicon transporter Lsi1 compromises inducibility of anti-herbivore defense in rice plants.

## Materials and Methods

### Plant Growth

Rice seeds of silicon-transporter deficient mutant *OsLsi1* and corresponding wild-type (WT, cv. Oochikara) were kindly provided by Dr. Jianfeng Ma of Okayama University (Japan) (Ma et al., 2002). Rice seeds were surface sterilized with 1% NaClO for 10 min, rinsed three times with sterile distilled water and then pre-imbibed in distilled water for 24 h at 28°C. After pre-germination for 3 days, the seedlings were hydroponically cultured in plastic pots (length × width × height, 35 × 25 × 12 cm) with support by a sponge. Each plastic pot contained 10 rice seedlings and 10 L of modified Kimura B nutrient solution [0.36 mM (NH_4_)_2_SO_4_, 0.36 mM Ca(NO_3_)_2_ ⋅ 4H_2_O, 0.27 mM K_2_SO_4_, 0.55 mM MgSO_4_ ⋅ 7H_2_O, 0.18 mM KH_2_PO_4_, 20 mM EDTA-Fe, 0.77 mM ZnSO_4_ ⋅ 7H_2_O, 0.32 mM CuSO_4_ ⋅ 5H_2_O, 46.26 mM H_3_BO_3_, 9.10 mM MnCl_2_, 0.15 mM (NH_4_)_6_Mo_7_O_24_ ⋅ 4H_2_O]. The nutrient solution was changed every 3 days throughout the experiment, and the pH was adjusted to 5.5 every day. The Si (NaSiO_3_ ⋅ 9H_2_O) concentration used in the experiment was 1.4 mM. For Si untreated plants, NaCl was also added to balance sodium levels. All seedlings were grown for additional 35 days in a greenhouse with day/night (14 h/10 h) temperature of 28°C/24°C, 75% relative humidity and natural sunlight. Each treatment had 20 pots with 10 plants per pot.

### LF Insects

*Cnaphalocrocis medinalis* Guenée (rice Leaffolder, LF) used for infestation and cultures was originally obtained from paddy fields on the campus of Fujian Agricultural and Forestry University in Fuzhou (China) and maintained on WT rice plants that have been growing for 25 days and kept in net cages in a greenhouse with the same conditions described above. The host plants were changed every 10 days. Larvae at the third-instar were used for all bioassays described.

### Experimental Design and Bioassays

There were four different experimental settings: (1) control WT plants without Si amendment (WT); (2) *OsLsi1* mutant rice plants without Si amendment (*OsLsi1*); (3) WT plants with Si (1.4 mM) amendment (WT+Si); (4) *OsLsi1* mutant plants with Si (1.4 mM) amendment (*OsLsi1* +Si). After rice plants had been cultured with the above four treatments for 35 days, 20 seedlings were randomly selected from each treatment group (*n* = 20), and each seedling was transplanted into a pot with 1 L of nutrient solution. Each seedling was individually infested with a third-instar LF larva that had been starved for 2 h and weighted before placement on leaves at node three (the youngest fully expanded leaf was defined as leaf 1). All larvae were weighed again 3 days after LF inoculation. The mass gain of larvae on each plant was calculated. These LF-inoculated and -uninoculated plants were separately placed on both sides of the greenhouse and separated with plastic sheets.

The leaves (leaf 3) and roots were harvested from LF-infested plants at 0, 3, 6, 12, 24, and 48 h after LF inoculation, immersed immediately in liquid nitrogen, and stored at −80°C for further analyses of MDA content, enzyme activity and relative expression of genes. Six plants were sampled for each treatment at each time point.

### Effects of Si Application on Rice Growth

Twenty seedlings of *OsLsi1* mutant and WT plants treated with or without Si as described above were randomly selected from each group and measured to calculate average shoot height and root length 35 days after transplantation. Plant height was measured from the stem base to the top of leaves, and root length was measured from the root base to the root tip.

### Effects of Si Application on Si Accumulation

The leaves and roots of *OsLsi1* mutant and WT rice plants cultured in Kimura B nutrient solution treated with or without Si as described above were separately harvested for analysis of Si content 35 days after transplantation. The Si content was analyzed by the colorimetric molybdenum blue method described by van der Vorm (1987). Si concentrations in the resulting solutions were measured at 811 nm using a spectrophotometer (Ye et al., 2013).

### Malondialdehyde Assay

Leaf and root samples (100 mg) were homogenized with 0.5 mL of 0.1% (w/v) trichloroacetic acid (TCA), the homogenate was centrifuged for 10 min and then the supernatant was aspirated. Malondialdehyde (MDA) was quantified by using the thiobarbituric acid method described by Mitsuru and Uchiyama (1978). All these measurements had six biologically replicates.

### Enzyme Activity Analysis

Catalase activity was estimated from the rate of H_2_O_2_ decomposition at 240 nm by the method established by Havir and Mchale (1989). SOD was extracted and assayed by the method described by Crapo et al. (1978). The PPO activity was determined using 0.05 M catechol as substrate, as described by Zauberman et al. (1991). The POD activity was measured using the guaiacol method as described by Kraus and Fletcher (1994). All these measurements were biologically repeated for six times.

### Quantitative Real-Time PCR Analysis

Procedures used for RNA extraction and reverse transcription of plant samples were described by Ye et al. (2013). Total RNA was extracted from 0.1 g flash-frozen, powdered leaf/root samples using the TRIzol^TM^ Reagent kit (Invitrogen, United States), according to the manufacturer’s instructions. Total RNA was treated with RNase-Free DNaseI (TIANGEN Biotech, Beijing) and 1 μg of total RNA was then pipetted for cDNA synthesis using the GoScript^TM^ Reverse Transcription System (Promega Biotech, Beijing). Real-time PCR was performed according to the procedure of UltraSYBR two-step fluorescence quantitative PCR kit (ComWin Biotech, Beijing). Reaction conditions for thermal cycling were 95°C for 60 s, followed by 40 cycles of 95°C for 20 s, 60°C for 15 s, then 72°C for 30 s. Fluorescence data were collected during the cycle at 60°C. Melting curve analysis and agarose gel electrophoresis were carried out to verify amplicon specificity. Calculation of gene expression level was normalized using the rice housekeeping gene *OsActin* and the 2^−ΔΔCT^ Method. Gene-specific primers used in this study are listed in Supplementary Table S1. All assays were performed in triplicate using three biological replicates per treatment.

### Data Analysis

The data were processed and plotted using Microsoft Excel 2013 and Origin 2018 software and the data were tested for significance by SPSS 19 statistical analysis software. The data for biomass, physiological and biochemical index were analyzed using a completely randomized design following a 2 × 2 × 2 factorial (two genotypes × two Si levels × two LF treatments) with three or six replicates. A two-way ANOVA and three-way ANOVA were used to test the significance of the treatment effect. If the interaction between the two factors or the three factors was significant, the Tukey test (*P* < 0.05) was performed to compare the differences between all treatments; If the interaction between the two is not significant, then a *t*-test was performed between different levels of each treatment. The experimental data are expressed as mean ± standard error.

## Results

### Si Effects on Rice Growth and Si Accumulation

**FIGURE 1:**
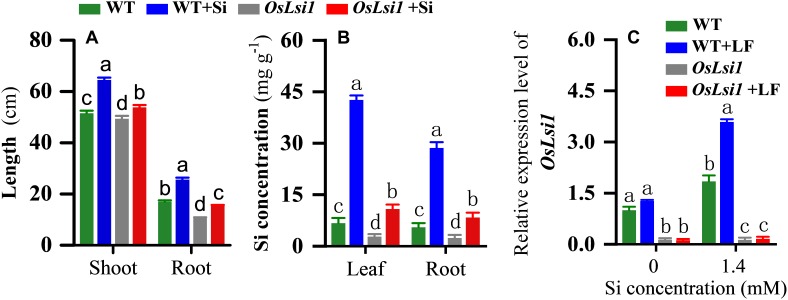
Effects of Si amendment on plant height and root length **(A)**, silicon accumulation **(B)** and transcript levels of *OsLsi1* **(C)** in rice plants of Si transporter deficient mutant *OsLsi1* and the corresponding wild type (WT). *OsLsi1* transcript levels were determined using RT-qPCR in the roots of WT and *OsLsi1* with Si-amendment and later infestation by rice leaffolder (LF). Values are mean ± standard error (SE) (*n* = 20 for plant height and root length, *n* = 6 for silicon content, *n* = 3 for transcript levels), letters above bars indicate significant difference among treatments (Tukey *post hoc* test, *P* < 0.05).

Addition of Si significantly increased the shoot length and root length of both WT and *OsLsi1* rice plants (Figure 1A). Si application enhanced the plant height and root length by 25.2 and 50.5% in WT plants, and 9.0 and 46.7% in *OsLsi1* plants, respectively (Figure 1A). Both shoot length and root length of WT plants were significantly greater than those of *OsLsi1* mutant.

In hydroponic culture very low concentrations of Si were detected in both WT and *OsLsi1* Si-untreated plants (Figure 1B). Si amendment significantly increased Si contents in the leaves and roots of both WT and *OsLsi1* plants. However, Si contents in Si-treated WT plants were much higher than those in Si-treated *OsLsi1* plants. The Si concentrations in the leaves and roots of Si-treated WT plants reached 42.62 and 28.61 mg/g, respectively, and these two values dropped to 10.82 and 8.36 mg/g in *OsLsi1* plants, respectively. A two-way ANOVA showed a significant interaction between genotype and Si treatment for plant height, root length and Si concentration in the leaf and root (Supplementary Table S2).

Real-time PCR analyses showed that in the absence of Si amendment, the *OsLsi1* mRNA expression was higher in WT roots than that in *OsLsi1* roots, and there was no significant difference between WT and WT+LF treatments (Figure 1C). Si addition upregulated transcript level of *OsLsi1* (1.84-fold) in WT plants, but it did not affect transcript level of *OsLsi1* in mutant *OsLsi1* plants (Figure 1C). Interestingly, LF infestation further upregulated transcript level of *OsLsi1* (1.95-fold) in Si-treated WT plants relative to un-infested plants.

### Si Effects on LF Performance

**FIGURE 2:**
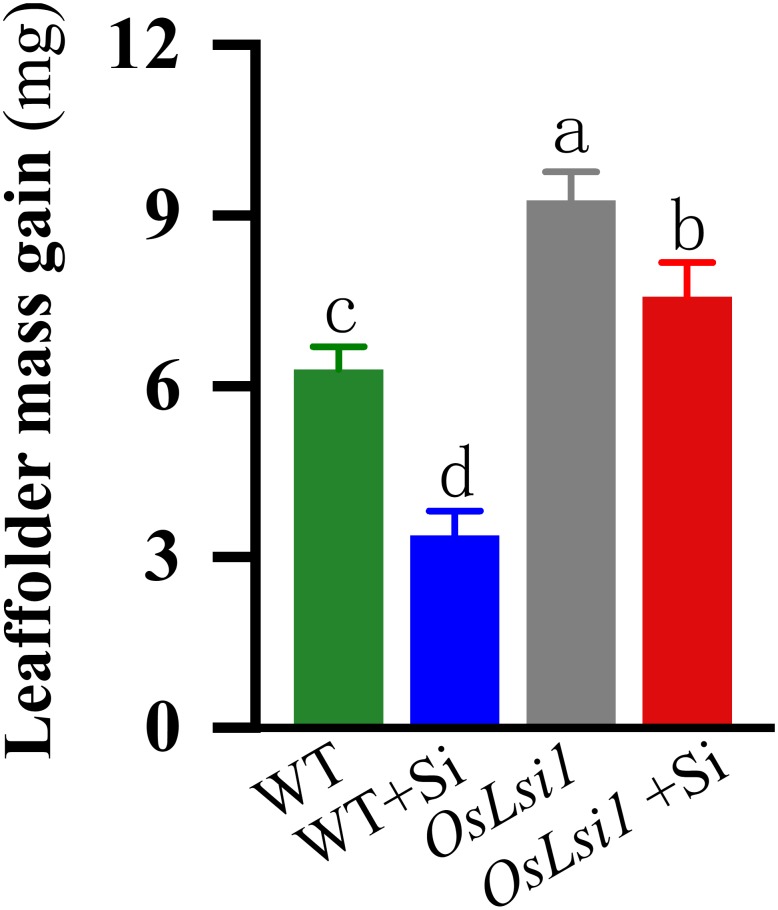
Mass gain of the third instars rice leaffolder (LF) fed on Si amended (+Si) and un-amended rice plants of Si transporter deficient mutant *OsLsi1* and the corresponding wild type (WT). Different genotypes of rice plants were fertilized with 0 or 1.4 mM silicon (Si) amendment for 30 days, and then treated by third-instar LF larvae that had been weighed and starved for 2 h. All larvae were weighed after 3 days of infestation, and the means of mass gain were then calculated. Values are mean ± SE (*n* = 20). Letters above bars indicate significant differences among treatments (Tukey’s multiple range test, *P* < 0.05).

Bioassays show that weight gain of LF larvae fed on WT plants increased 6.30 mg 3 days after insect infestation, whereas larvae fed on Si-treated WT plants increased only 3.38 mg (Figure 2), indicating that Si addition significantly enhanced WT resistance against LF (Supplementary Table S2). LF larvae fed on Si-untreated and Si-treated *OsLsi1* mutant gained mass by 9.27 and 7.58 mg, respectively.

### Si Effects on Malondialdehyde (MDA)

**FIGURE 3:**
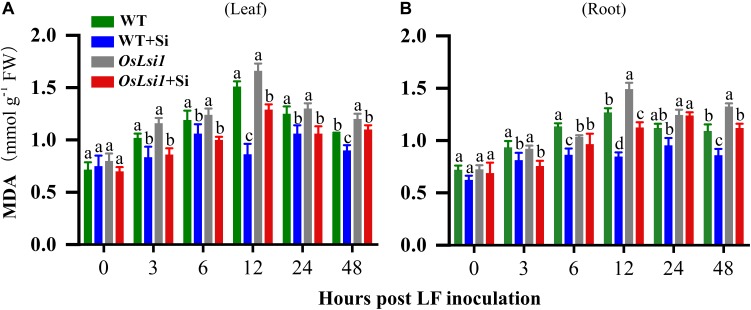
Concentrations of malondialdehyde (MDA) in the leaves **(A)** and roots **(B)** of Si amended and un-amended rice plants of Si transporter deficient mutant *OsLsi1* and the corresponding wild type (WT) at 0, 3, 6, 12, 24, and 48 h post-inoculation with the third instar larvae of rice leaffolder (LF). Si and LF treatments were performed as described for experiments shown in Figure 1, 2. Values are mean ± SE (*n* = 6). For each time point, letters above bars indicate significant difference among treatments (Tukey’s multiple range test, *P* < 0.05).

After LF infestation of the leaves of rice plants for 48 h, MDA contents in the leaves and roots were examined. Before LF inoculation (time point 0) MDA contents did not display any significant difference among four treatments in both leaves and roots (Figure 3A,B). Thereafter, MDA contents in the leaves and roots of both WT and *OsLsi1* plants gradually increased up to 12 h after LF inoculation, reached 1.51 and 1.66 mmol/g FW in leaves, respectively, and reached 1.27 and 1.49 mmol/g FW in roots, respectively. In contrast, Si amendment significantly reduced MDA contents in the leaves and roots of both WT and *OsLsi1* plants. In WT plants Si amendment reduced MDA by 43.0 and 33.1% in the leaves and roots, respectively, at 12 h after LF infestation. MDA contents in both leaves and roots were lower in Si-treated WT plants relative to Si-treated *OsLsi1* plants. There were no significant interactions between effects of genotype and Si treatment on MDA contents (Supplementary Table S3).

### Silicon-Enhanced Resistance by Inducing Defense-Related Enzymes

**FIGURE 4:**
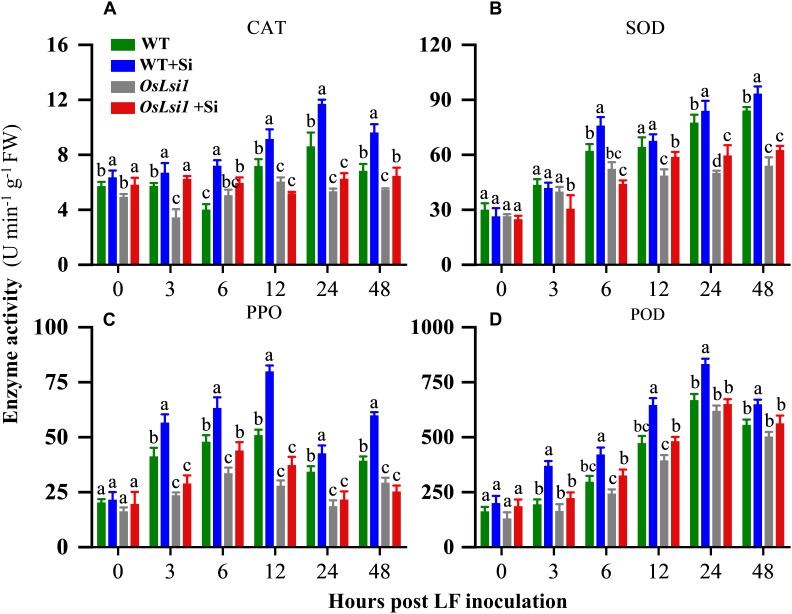
Activity of **(A)** catalase (CAT), **(B)** superoxide dismutase (SOD), **(C)** polyphenol oxidase (PPO) and **(D)** peroxidase (POD) in the leaves of Si amended and un-amended rice plants after inoculation with third-instar LF larvae. Si and LF treatments were performed as described for experiments shown in Figure 1, 2. Values are mean ± SE (*n* = 6). For each time point, letters above bars indicate significant difference among treatments (Tukey’s multiple range test, *P* < 0.05).

**FIGURE 5:**
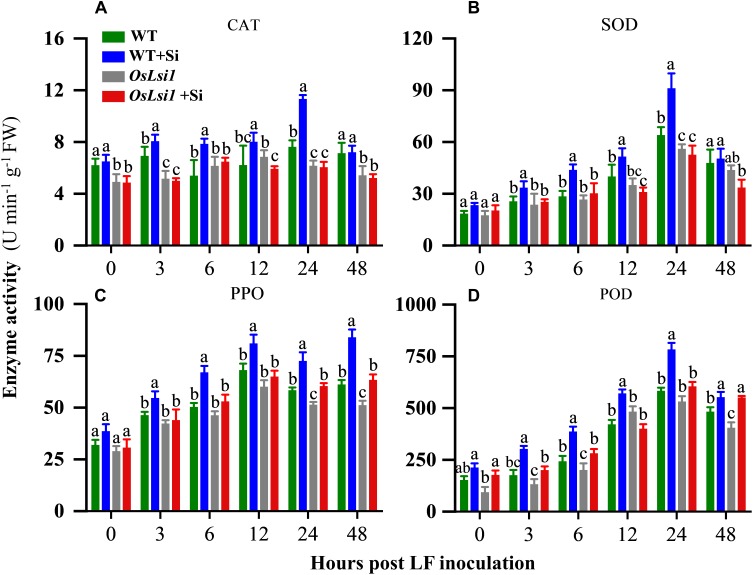
Activity of **(A)** catalase (CAT), **(B)** superoxide dismutase (SOD), **(C)** polyphenol oxidase (PPO) and **(D)** peroxidase (POD) in the roots of Si amended and un-amended rice plants after inoculation with third-instar LF larvae. Si and LF treatments were performed as described for experiments shown in Figure 1, 2. Values are mean ± SE (*n* = 6). For each time point, letters above bars indicate significant difference among treatments (Tukey’s multiple range test, *P* < 0.05).

Four defense-related enzymes, including catalase (CAT), superoxide dismutase (SOD), polyphenol oxidase (PPO), and peroxidase (POD) were analyzed to compare different effects of Si amendment on their activities in the leaves and roots of WT and *OsLsi1* plants. Si amendment alone did not significantly alter activities of SOD, PPO and POD in both WT and *OsLsi1* rice plants (Figure 4, 5). However, after LF infestation the Si amendment significantly enhanced activities of CAT, SOD, PPO, and POD in both leaves and roots of WT plants (Supplementary Tables S5, S6). Although insect herbivory induced these defense-related enzymes in *OsLsi1* mutant plants, the magnitude of induction was higher in Si-treated WT plants. After 3–6 h of LF infestation, the activities of CAT, SOD, PPO, and POD in WT+Si plants were rapidly activated, this effect was particularly evident at 12–48 h after LF infestation (Figure 4, 5). For example, in absence of LF, levels of CAT, SOD, PPO, and POD in the leaves increased by 27.3, 5.2, 56.8, and 36.5% at 12 h, and increased by 35.8, 8.2, 24.3, and 24.6% at 24 h after LF infestation, respectively, in Si-treated WT seedlings relative to Si untreated seedlings (Figure 4). By contrast, in presence of LF infestation, CAT, SOD, PPO, and POD activities in the leaves increased by 51.0, 38.9, 63.7, and 186.0% at 12 h, and by 118.9, 67.3, 34.3, and 128.6% at 24 h, respectively. In the WT plants, the activity of CAT in leaves was also significantly higher than the CAT activity in leaves of Si-treated and untreated *OsLsi1* at 12 and 24 h after LF infestation (Figure 4 and Supplementary Table S5). Similarly, SOD, PPO, and POD also showed similar trends in the leaves. In the roots, the activities of the four defense enzymes in the WT+Si plants were also induced more rapidly and strongly by LF infestation, especially at 24 h after LF infestation (Figure 5 and Supplementary Table S6).

### Silicon-Enhanced Resistance by Inducing Jasmonate-Dependent Genes

**FIGURE 6:**
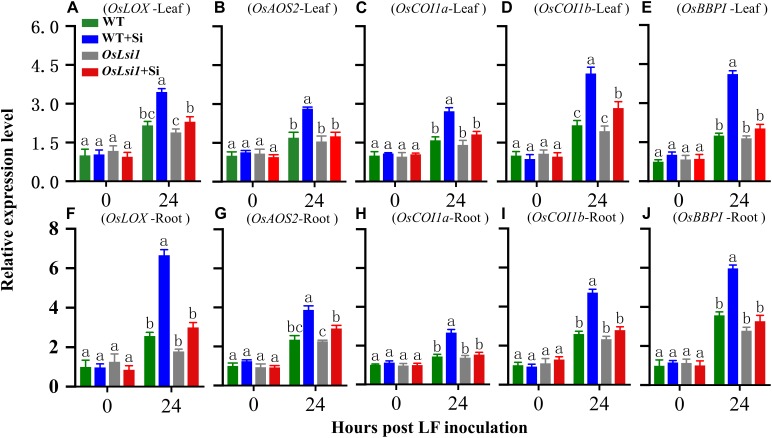
Transcript levels of *OsLOX* **(A,F)**, *OsAOS2* **(B,G)**, *OsCOI1a* **(C,H)**, *OsCOI1b* **(D,I)**, and *OsBBPI* **(E,J)** in the leaves and roots of Si amended and un-amended rice plants after inoculation with third-instar LF larvae. Si and LF treatments were performed as described for experiments shown in Figure 1, 2. Values are mean ± SE (*n* = 3). Letters above bars indicate significant difference among treatments (Tukey’s multiple range test, *P* < 0.05).

The jasmonate (JA) signaling pathway plays a central role in mediating plant defense responses against insect herbivores (Howe and Jander, 2008). Protease inhibitors such as Bowman-Birk protease inhibitor (BBPI) are important components of plant inducible defense responses (Rakwal et al., 2001). Expression levels for *OsLOX*, *OsAOS*2, *OsCOI1a*, *OsCOI1b* in the JA pathway and *OsBBPI* were monitored in the leaves and roots of the two genotypes with or without Si amendment 24 h after LF inoculation (Figure 6). Real-time RT-PCR analyses showed that all tested genes were induced by LF feeding as expected. However, the highest transcript levels of all five tested genes were found in both leaves and roots of Si-treated WT plants. A three-way ANOVA showed a significant interaction between the three factors (genotype × Si treatment × LF treatment), for *OsCOI1a* (*P* = 0.016) and *OsBBPI* (*P* = 0.025) in the leaves, for *OsLOX* (*P* = 0.034), and *OsCOI1b* (*P* = 0.047) in the roots (Supplementary Table S4). Compared with Si-untreated WT plants, transcript levels of *OsLOX*, *OsAOS*2, *OsCOI1a*, *OsCOI1b*, and *OsBBPI* were induced 1.60-, 1.66-, 1.70-, 1.92-, and 2.35-fold in the leaves (Figure 6A–E), and 2.60-, 1.65-, 1.85-, 1.82-, and 1.68-fold in the roots (Figure 6F–J), respectively, in Si-treated WT plants after insect herbivory. However, deficiency in silicon transporter Lsi1 led to reduced induction of the transcripts of *OsLOX*, *OsAOS*2, *OsCOI1a*, *OsCOI1b*, and *OsBBPI* by Si in response to LF herbivory. Only *OsLOX* and *OsCOI1b* were obviously induced by Si in the leaves in *OsLsi1* mutant plants, suggesting that deficiency in silicon transporter Lsi1 impairs inducibility of rice defense against insect pests.

## Discussion

The beneficial effects of silicon in enhancing plant resistance to biotic and abiotic stresses have been observed in a wide range plant species (Ma et al., 2004). Rice is a Si accumulator with up to 10% Si on a dry weight basis in the shoot (Ma and Yamaji, 2006). Si accumulates in plant tissues, which could act as a physical barrier to reduce digestibility of plant tissues by herbivore insects. Recently, induced defense has been shown to be a mechanism of Si-enhanced plant resistance (Ye et al., 2013).

Lsi1 is an aquaporin-like transmembrane protein and a root-specific Si transporter responsible for active transport of Si from the soil solution to the root cells of rice (Ma et al., 2006; Yamaji et al., 2015). By using Si transporter deficient mutant *OsLsi1* and the corresponding WT rice plants we show that deficiency in Si transporter Lsi1 led to reduced Si accumulation in both leaves and roots, particularly after Si amendment (Figure 1B). Interestingly, Lsi1 deficiency resulted in reduced root and shoot growth (Figure 1A,C). Si amendment promoted the growth of both roots and shoots of rice plants, which is consistent with other findings (Ma and Takahashi, 1990; Hossain et al., 2002).

Furthermore, Si amendment on WT plants greatly reduced LF larval weight (Figure 2), which is consistent with other recent findings (Ye et al., 2013; Han et al., 2015) showing that Si application significantly delayed LF larval development and reduced larval survival rate and pupation rate. However, Si application on *OsLsi1* mutant plants showed a reduced effect on LF performance. The plant resistance level in Si-treated *OsLsi1* mutant was even lower (as reflected in LF mass gain) than that in Si-untreated WT plants (Figure 2), suggesting that Si effect on rice anti-herbivore resistance was significantly attenuated by Si transporter deficiency.

Herbivory by chewing insects causes various mechanical structural damage, thereby accelerating the process of membrane lipid peroxidation and MDA accumulation. Therefore, MDA content is an indicator reflecting the membrane lipid peroxidation of plants and the extent of reactive oxygen species (ROS) damage (Bhattacharjee, 2005). MDA contents in the leaves and roots of Si-pretreated plants were significantly lower after LF infestation than those in Si-untreated plants (Figure 3). Although Si amendment reduced the MDA content in the leaves and roots of *OsLsi1* plants after LF infestation, the MDA content in Si-treated WT was significantly lower than that in Si-treated *OsLsi1*. The result indicates that Si addition can protect plants against insect herbivores by lowering membrane lipid peroxidation and oxidative damage.

jasmonate is known to play a central role in mediating plant defense responses against insect herbivores (Howe and Jander, 2008). Our real-time RT-PCR analyses showed that the expression levels of JA dependent genes including *OsLOX*, *OsAOS2*, *OsCOI1a*, *OsCOI1b*, and *OsBBPI* were much higher in Si-treated WT plants after LF infestation than those in other treatments (Figure 6). All five tested defense-related genes were induced 24 h after LF infestation in Si-treated WT plants, while no or lower induction was found at this time point in Si-untreated WT plants and Si-treated or un-treated Si-transporter deficient plants. Si application alone did not show any effect on expression levels of the five genes in rice plants, suggesting that Si application amplifies the induced defense responses upon insect attack. Such amplified effect of Si on induced defense responses has also been observed in plant response to pathogen attack. Transcriptome analyses by Fauteux et al. (2006) and Chain et al. (2009) showed that Si treatment alone only induced a few genes in the absence of pathogens, while it affected transcription of many genes in pathogen-inoculated plants. Our previous work showed that Si-treated rice plants showed significantly higher induction of defense-related enzymes including POD, PPO, and phenylalanine ammonia-lyase (PAL) in rice response to infection by blast pathogen *Magnaporthe grisea* (Cai et al., 2008).

Furthermore, *OsLsi1* transcript level was much higher in WT plants, especially after LF infestation (Figure 1C). However, there was no difference in *OsLsi1* transcript level in *OsLsi1* mutant plant before and after Si application and LF infestation (Figure 1C). These results reveal that Si absorption can be induced by insect herbivory, which is consistent with our previous finding (Ye et al., 2013).

Jasmonate and Silicon have been well documented to be involved in rice resistance to insect herbivores (Reynolds et al., 2009, 2016). Our previous study showed strong interaction between JA and Si in rice defense against insect herbivores (Ye et al., 2013). The results here further confirm the interaction between JA and Si, in which Si induces JA dependent defense responses to herbivory, including the elevated induction of defense-related enzymes (Figure 4, 5) and enhanced induction of transcripts encoding proteins involved in JA signaling (Figure 6) following insect attack. However, significant decreases in activity of defense-related enzymes and transcript levels of JA dependent genes, and an apparent loss of Si-induced LF resistance were observed in Si transporter deficient mutant *OsLsi1*, regardless of Si treatment. Therefore, rice, and likely other plants accumulating high levels of Si, may have evolved Si-mediated defense mechanisms in which Si is an integrated component and deficiency in Si transporters may compromise inducibility of anti-herbivore defense in these plants.

## Author Contributions

YL, YS, and RZ designed the research. YL, ZS, RX, WC, and SS performed the research. YL, YS, and ZL analyzed the data. YL, YS, TL, and RZ wrote the manuscript.

## Conflict of Interest Statement

The authors declare that the research was conducted in the absence of any commercial or financial relationships that could be construed as a potential conflict of interest.

## References

[B1] Bhattacharjee S. (2005). Reactive oxygen species and oxidative burst: roles in stress, senescence and signal transduction in plants. *Curr. Sci.* 89 1113–1121.

[B2] Cai K. Gao D. Luo S. Zeng R. Yang J. Zhu X. (2008). Physiological and cytological mechanisms of silicon-induced resistance in rice against blast disease. *Physiol. Plant.* 134 324–333. 10.1111/j.1399-3054.2008.01140.x 18513376

[B3] Chain F. Côté-Beaulieu C. Belzile F. Menzies J. G. Bélanger R. R. (2009). A comprehensive transcriptomic analysis of the effect of silicon on wheat plants under control and pathogen stress conditions. *Mol. Plant Microbe Infect.* 22 1323–1330. 10.1094/mpmi-22-11-1323 19810802

[B4] Chen D. M. Chen D. Q. Xue R. R. Long J. Lin X. H. Lin Y. B. (2019). Effects of boron, silicon and their interactions on cadmium accumulation and toxicity in rice plants. *J. Hazard. Mater.* 367 447–455. 10.1016/j.jhazmat.2018.12.111 30611037

[B5] Crapo J. D. Mccord J. M. Fridovich I. (1978). Preparation and assay of superoxide dismutases. *Methods Enzymol.* 53 382–393. 10.1016/S0076-6879(78)53044-9 362127

[B6] Devrim C. Britto D. T. Huynh W. Q. Herbert J. K. (2016). The role of silicon in higher plants under salinity and drought stress. *Front. Plant Sci.* 7:1072. 10.3389/fpls.2016.01072 27486474 PMC4947951

[B7] Fauteux F. Chain F. Belzile F. Menzies J. G. Bélanger R. R. (2006). The protective role of silicon in the Arabidopsis-powdery mildew pathosystem. *Proc. Natl. Acad. Sci. U.S.A.* 103 17554–17559. 10.1073/pnas.0606330103 17082308 PMC1859967

[B8] Ghareeb H. Bozsó Z. Ott P. G. Repenning C. Stahl F. Wydra K. (2011). Transcriptome of silicon-induced resistance against *Ralstonia solanacearum* in the silicon non-accumulator tomato implicates priming effect. *Physiol. Mol. Plant Pathol.* 75 83–89. 10.1016/j.pmpp.2010.11.004

[B9] Glare T. Caradus J. R. Gelernter W. Jackson T. A. Keyhani N. O. Köhl J. (2012). Have biopesticides come of age? *Trends Biotechnol.* 30 250–258. 10.1016/j.tibtech.2012.01.003 22336383

[B10] Godfray H. C. J. Beddington J. R. Crute I. R. Haddad L. Lawrence D. Muir J. F. (2010). Food security: the challenge of feeding 9 billion people. *Science* 327 812–818. 10.1126/science.1185383 20110467

[B11] Guntzer F. Keller C. Meunier J. D. (2012). Benefits of plant silicon for crops: a review. *Agron. Sustain. Dev.* 32 201–213. 10.1007/s13593-011-0039-8

[B12] Han Y. Q. Lei W. B. Wen L. Z. Hou M. L. (2015). Silicon-mediated resistance in a susceptible rice variety to the rice leaf folder, *Cnaphalocrocis medinalis* Guenée (Lepidoptera: Pyralidae). *PLoS One* 10:e0120557. 10.1371/journal.pone.0120557 25837635 PMC4383528

[B13] Havir E. A. Mchale N. A. (1989). Enhanced-peroxidatic activity in specific catalase isozymes of tobacco, barley, and maize. *Plant Physiol.* 91 812–815. 10.2307/4272432 16667141 PMC1062080

[B14] Hossain M. T. Mori R. Soga K. Wakabayashi K. Kamisaka S. Fujii S. (2002). Growth promotion and an increase in cell wall extensibility by silicon in rice and some other Poaceae seedlings. *J. Plant Res.* 115 0023–0027. 10.1007/s102650200004 12884045

[B15] Howe G. A. Jander G. (2008). Plant immunity to insect herbivores. *Annu. Rev. Plant Biol.* 59 41–66. 10.1146/annurev.arplant.59.032607.092825 18031220

[B16] Keeping M. G. Miles N. Sewpersad C. (2014). Silicon reduces impact of plant nitrogen in promoting stalk borer (*Eldana saccharina*) but not sugarcane thrips (*Fulmekiola serrata*) infestations in sugarcane. *Front. Plant Sci.* 5:289. 10.3389/fpls.2014.00289 24999349 PMC4064666

[B17] Kraus T. E. Fletcher R. A. (1994). Paclobutrazol protects wheat seedlings from heat and paraquat injury. is detoxification of active oxygen involved? *Plant Cell Physiol.* 35 45–52. 10.1093/oxfordjournals.pcp.a078569

[B18] Kvedaras O. L. An M. Choi Y. S. Gurr G. M. (2010). Silicon enhances natural enemy attraction and biological control through induced plant defences. *Bull. Entomol. Res.* 100 367–371. 10.1017/S0007485309990265 19737442

[B19] Liang Y. C. Nikolic M. Bélanger R. Gong H. J. Song A. L. (2015). *Silicon in Agriculture.* Dordrecht: Springer.

[B20] Liu J. Zhu J. W. Zhang P. J. Han L. W. Reynolds O. L. Zeng R. S. (2017). Silicon supplementation alters the composition of herbivore induced plant volatiles and enhances attraction of parasitoids to infested rice plants. *Front. Plant Sci.* 8:1265. 10.3389/fpls.2017.01265 28769965 PMC5515826

[B21] Ma J. F. Mitani N. Nagao S. Konishi S. Tamai K. Iwashita T. (2004). Characterization of the silicon uptake system and molecular mapping of the silicon transporter gene in rice. *Plant Physiol.* 136 3284–3289. 10.1104/pp.104.047365 15448199 PMC523387

[B22] Ma J. F. Takahashi E. (1990). Effect of silicon on the growth and phosphorus uptake of rice. *Plant Soil* 126 115–119. 10.1007/bf00041376

[B23] Ma J. F. Takahashi E. (1991). Effect of silicate on phosphate availability for rice in a P-deficient soil. *Plant Soil* 133 151–155. 10.1007/BF00009187

[B24] Ma J. F. Takahashi E. (2002). *Soil, Fertilizer, and Plant Silicon Research in Japan.* Amsterdam: Elsevier Science.

[B25] Ma J. F. Tamai K. Ichii M. Wu G. F. (2002). A rice mutant defective in Si uptake. *Plant Physiol.* 130 2111–2117. 10.1104/pp.010348 12481095 PMC166723

[B26] Ma J. F. Tamai K. Yamaji N. Mitani N. Konishi S. Katsuhara M. (2006). A silicon transporter in rice. *Nature* 440 688–691. 10.1038/nature04590 16572174

[B27] Ma J. F. Yamaji N. (2006). Silicon uptake and accumulation in higher plants. *Trends Plant Sci.* 11 390–397. 10.1016/j.tplants.2006.06.007 16839801

[B28] Markovich O. Steiner E. Kouřil Š Tarkowski P. Aharoni A. Elbaum R. (2017). Silicon promotes cytokinin biosynthesis and delays senescence in *Arabidopsis* and Sorghum. *Plant Cell Environ.* 40 1189–1196. 10.1111/pce.12913 28102542

[B29] Massey F. P. Ennos A. R. Hartley S. E. (2006). Silica in grasses as a defence against insect herbivores: contrasting effects on folivores and a phloem feeder. *J. Anim. Ecol.* 75 595–603. 10.1111/j.1365-2656.2006.01082.x 16638012

[B30] Massey F. P. Hartley S. E. (2009). Physical defences wear you down: progressive and irreversible impacts of silica on insect herbivores. *J. Anim. Ecol.* 78 281–291. 10.1111/j.1365-2656.2008.01472.x 18771503

[B31] Meena V. D. Dotaniya M. L. Coumar V. Rajendiran S. Kundu S. Rao A. S. (2014). A case for silicon fertilization to improve crop yields in tropical soils. *Proc. Natl. Acad. Sci. India B Biol Sci.* 84 505–518.

[B32] Meharg C. Meharg A. A. (2015). Silicon, the silver bullet for mitigating biotic and abiotic stress, and improving grain quality, in rice? *Environ. Exp. Bot*. 120 8–17. 10.1016/j.envexpbot.2015.07.001

[B33] Mitsuru M. Uchiyama M. (1978). Determination of malonaldehyde precursor in tissues by thiobarbituric acid test. *Anal. Biochem.* 86 271–278. 10.1016/0003-2697(78)90342-1 655387

[B34] Rakwal R. Agrawal G. K. Jwa N. S. (2001). Characterization of a rice (*Oryza sativa* L.) Bowman-Birk proteinase inhibitor: Tightly light regulated induction in response to cut, jasmonic acid, ethylene and protein phosphatase 2A inhibitors. *Gene* 263 189–198. 10.1016/S0378-1119(00)00573-4 11223257

[B35] Reynolds O. L. Keeping M. G. Meyer J. H. (2009). Silicon-augmented resistance of plants to herbivorous insects: a review. *Ann. Appl. Biol.* 155 171–186. 10.1111/j.1744-7348.2009.00348.x

[B36] Reynolds O. L. Padula M. P. Zeng R. S. Gurr G. M. (2016). Silicon: potential to promote direct and indirect effects on plant defense against arthropod pests in agriculture. *Front. Plant Sci.* 7:744. 10.3389/fpls.2016.00744 27379104 PMC4904004

[B37] Richmond K. E. Sussman M. (2003). Got silicon? The non-essential beneficial plant nutrient. *Curr. Opin. Plant Biol.* 6 268–272. 10.1016/S1369-5266(03)00041-4 12753977

[B38] Rios J. J. Martínez-Ballesta M. C. Ruiz J. M. Leon M. B. B. Carvajal M. (2017). Silicon-mediated improvement in plant salinity tolerance: the role of aquaporins. *Front. Plant Sci.* 8:948. 10.3389/fpls.2017.00948 28642767 PMC5463179

[B39] Rockstrom J. Steffen W. Noone K. J. Persson Å Chapin F. S. III Lambin E. F. (2009). Planetary boundaries: Exploring the safe operating space for humanity. *Ecol. Soc.* 14:32. 10.1890/07-0978.1 25679962

[B40] Salim M. Saxena R. C. (1992). Iron, silica, and aluminum stresses and varietal resistance in rice: effects on whitebacked planthopper. *Crop Sci.* 32 212–219. 10.2135/cropsci1992.0011183X003200010044x

[B41] Sanchis V. Bourguet D. (2008). *Bacillus thuringiensis*: applications in agriculture and insect resistance management. A review. *Agron. Sustain. Dev.* 28 11–20. 10.1051/agro:2007054

[B42] Sasamoto K. (1958). Studies on the relation between the silica content in the rice plant and the insect pests. Part 6. *Jpn. J. Appl. Entomol. Z* 2 88–92.

[B43] Savant N. K. (1997). Silicon management and sustainable rice production. *Adv. Agron.* 58 151–199. 10.1016/S0065-2113(08)60255-2

[B44] Sommer M. Kaczorek D. Kuzyakov Y. Breuer J. (2006). Silicon pools and fluxes in soils and landscapes-a review. *J. Plant Nutr. Soil Sci.* 169 310–329. 10.1002/jpln.200521981

[B45] Van Bockhaven J. Spíchal L. Novák O. Strnad M. Asano T. Kikuchi S. (2015). Silicon induces resistance to the brown spot fungus *Cochliobolus miyabeanus* by preventing the pathogen from hijacking the rice ethylene pathway. *New Phytol.* 206 761–773. 10.1111/nph.13270 25625327

[B46] van der Vorm P. D. J. (1987). Dry ashing of plant material and dissolution of the ash in HF for the colorimetric determination of silicon. *Commun. Soil Sci. Plant* 18 1181–1189. 10.1080/00103628709367893

[B47] Wang M. Gao L. M. Dong S. Y. Sun Y. M. Shen Q. R. Guo S. W. (2017). Role of silicon on plant-pathogen interactions. *Front. Plant Sci.* 8:701. 10.3389/fpls.2017.00701 28529517 PMC5418358

[B48] Wu K. M. Guo Y. Y. (2005). The evolution of cotton pest management practices in China. *Annu. Rev. Entomol.* 50 31–52. 15355239 10.1146/annurev.ento.50.071803.130349

[B49] Yamaji N. Ma J. F. (2007). Spatial distribution and temporal variation of the rice silicon transporter Lsi1. *Plant Physiol.* 143 1306–1313. 10.1104/pp.106.093005 17259286 PMC1820904

[B50] Yamaji N. Ma J. F. (2008). A transporter regulating silicon distribution in rice shoots. *Plant Cell* 20 1381–1389. 10.1105/tpc.108.059311 18515498 PMC2438455

[B51] Yamaji N. Ma J. F. (2011). Further characterization of a rice Si efflux transporter, Lsi2. *Soil Sci. Plant Nutr.* 57 259–264. 10.1080/00380768.2011.565480

[B52] Yamaji N. Sakurai G. Mitani-Ueno N. Ma J. F. (2015). Orchestration of three transporters and distinct vascular structures in node for intervascular transfer of silicon in rice. *Proc. Natl. Acad. Sci. U.S.A.* 112 11401–11406. 10.1073/pnas.1508987112 26283388 PMC4568664

[B53] Ye M. Song Y. Y. Long J. Wang R. L. Baerson S. R. Pan Z. Q. (2013). Priming of jasmonate-mediated antiherbivore defense responses in rice by silicon. *Proc. Natl. Acad. Sci. U.S.A.* 110 E3631–E3639. 10.1073/pnas.1305848110 24003150 PMC3780902

[B54] Zauberman G. Ronen R. Akerman M. Weksler A. Rot I. Fuchs Y. (1991). Post-harvest retention of the red colour of litchi fruit pericarp. *Sci. Hortic.* 47 89–97. 10.1016/0304-4238(91)90030-3 23572609

