# Supplementary material for: Deficiency in Silicon Transporter Lsi1 Compromises Inducibility of Anti-herbivore Defense in Rice Plants
Source: Front Plant Sci. 2019 May 24;10:652. doi: 10.3389/fpls.2019.00652 (PMC6543919; doi:10.3389/fpls.2019.00652)
Supplement: Supplementary file 1 [file Data_Sheet_1.docx]

**TABLE S1．** Gene-speciﬁc primers for quantitative RT-PCR

| Gene | Accession NO. | Specific Primer |
| --- | --- | --- |
| *OsLOX2* | UI3681 | F:5’-ATCTCCCAAGTGAAACACCACA-3 |
|  |  | R:5’-TCATAAACCCTGTCCCATTCTTC-3’ |
| *OsAOS2* | 03g12500 | F:5’-CTCGTCGGAAGGCTGTTGCT -3’ |
|  |  | R:5’-ACGATTGACGGCGGAGGTT -3’ |
| *OsActin* | DQ159948 | F:5’-CTGCGGGTATCCATGAGACT -3’ |
|  |  | R:5’-GCAATGCCAGGGAACATAGT-3’ |
| *OsLsi1* | 02g0745100 | F:5’-TCGCCGACTTCTTCCCTC -3’ |
|  |  | R:5’-ATCGCTCCGGTGAACTGC -3’ |
| *OsCOI1a* | AY423550 | F:5’-GATGCCCTCCCTGAGATACA -3’ |
|  |  | R:5’-AGTCAGACCTCCTTCCAGCA -3’ |
| *OsCOI1b* | AY423551 | F:5’-TCTGGGTAATGTTGGGGAAA -3’ |
|  |  | R:5’-GCCCTGTACCCACACGTATC -3’ |
| *OsBBPI* | U76004 | F: 5'-GCTCATCTGCGAGGACATCT-3' |
|  |  | R: 5'-TTCCTCATGGTCCACACAAG-3' |

**TABLE S2．** Two-way ANOVA of rice growth length, Si concentration and LF mass gain as influenced by genotype and Si treatment

| Items | Part | G | Si | G × Si |
| --- | --- | --- | --- | --- |
| Length | Shoot | < 0.001*** | < 0.001*** | < 0.001*** |
|  | Root | < 0.001*** | < 0.001*** | < 0.001*** |
| Si concentration | Leaf | < 0.001*** | < 0.001*** | < 0.001*** |
|  | Root | < 0.001*** | < 0.001*** | 0.001** |
| LF mass gain | | < 0.001*** | < 0.001*** | 0.182 |

*, *P* < 0.05; **, *P* < 0.01, ***, *P* < 0.001. G represents Genotype; Si represents Silicon treatment.

**TABLE S3．** Two-way ANOVA of MDA content in rice as influenced by genotype and Si treatment

| Part | Source | Time Points (h) | | | | | |
| --- | --- | --- | --- | --- | --- | --- | --- |
|  |  | 0 | 3 | 6 | 12 | 24 | 48 |
| Leaf | G | 0.859 | 0.344 | 0.821 | 0.010* | 0.767 | 0.014* |
|  | Si | 0.643 | 0.011* | 0.046* | < 0.001*** | 0.023* | 0.018* |
|  | G × Si | 0.416 | 0.442 | 0.442 | 0.110 | 0.921 | 0.721 |
| Root | G | 0.579 | 0.558 | 0.990 | < 0.001*** | < 0.001*** | < 0.001*** |
|  | Si | 0.350 | 0.025* | 0.013* | < 0.001*** | 0.113 | 0.001** |
|  | G × Si | 0.646 | 0.795 | 0.118 | 0.600 | 0.134 | 0.902 |

*, *P* < 0.05; **, *P* < 0.01, ***, *P* < 0.001. G represents Genotype; Si represents Silicon treatment.

**TABLE S4．** Three-way ANOVA of expression of insect defense related genes in rice as influenced by genotype，Si treatment and LF treatment

| Part | Genes | G | Si | I | G × Si | G × I | Si × I | G × Si × I |
| --- | --- | --- | --- | --- | --- | --- | --- | --- |
| Leaf | *OsLOX* | < 0.001*** | 0.017* | 0.002** | 0.015* | 0.031* | 0.022* | 0.096 |
|  | *OsAOS2* | 0.168 | 0.123 | 0.012* | 0.222 | 0.252 | 0.034* | 0.339 |
|  | *OsCOI1a* | 0.098 | 0.009** | 0.044* | 0.396 | 0.071 | 0.037* | 0.016* |
|  | *OsCOI1b* | 0.121 | 0.021* | 0.010* | 0.237 | 0.006** | 0.017* | 0.217 |
|  | *OsBBPI* | 0.017* | 0.012* | 0.005** | 0.001** | 0.068 | 0.003** | 0.025* |
| Root | *OsLOX* | 0.028* | 0.003** | 0.017* | 0.008** | 0.004** | 0.073 | 0.034* |
|  | *OsAOS2* | 0.035* | 0.011* | 0.010* | 0.022* | 0.32 | 0.038* | 0.397 |
|  | *OsCOI1a* | 0.013* | 0.110 | 0.016* | 0.151 | 0.005** | 0.026* | 0.119 |
|  | *OsCOI1b* | 0.204 | 0.054 | 0.001** | 0.219 | 0.012* | 0.018* | 0.047* |
|  | *OsBBPI* | 0.078 | 0.032* | 0.002** | 0.273 | 0.007** | 0.016* | 0.052 |
|  | *OsLsi1* | < 0.001*** | 0.017* | 0.002** | 0.015* | 0.031* | 0.022* | 0.096 |

*, *P* < 0.05; **, *P* < 0.01, ***, *P* < 0.001. G represents Genotype; Si represents Silicon treatment; I represents LF treatment.

**TABLE S5．** Two-way ANOVA of enzyme activities in rice leaves as influenced by genotype and Si treatment

| Enzymes | Source | Time Points (h) | | | | | |
| --- | --- | --- | --- | --- | --- | --- | --- |
|  |  | 0 | 3 | 6 | 12 | 24 | 48 |
| CAT | G | 0.226 | 0.037* | 0.945 | 0.001** | < 0.001*** | 0.004** |
|  | Si | 0.149 | 0.010* | 0.003** | 0.231 | 0.007** | 0.012* |
|  | G × Si | 0.973 | 0.104 | 0.043* | 0.016* | 0.075 | 0.162 |
| SOD | G | 0.388 | 0.158 | 0.001** | 0.027* | 0.001** | < 0.001*** |
|  | Si | 0.467 | 0.274 | 0.480 | 0.210 | 0.202 | 0.080 |
|  | G × Si | 0.771 | 0.406 | 0.043* | 0.582 | 0.680 | 0.959 |
| POD | G | 0.524 | 0.021* | 0.031* | 0.005** | 0.007** | 0.069 |
|  | Si | 0.225 | 0.003** | 0.008** | 0.003** | 0.013* | 0.029* |
|  | G × Si | 0.787 | 0.095 | 0.381 | 0.180 | 0.051 | 0.854 |
| PPO | G | 0.400 | 0.001** | 0.004** | < 0.001*** | 0.001** | < 0.001*** |
|  | Si | 0.530 | 0.032* | 0.016* | 0.001** | 0.141 | 0.006** |
|  | G × Si | 0.897 | 0.269 | 0.522 | 0.027* | 0.365 | 0.001** |

*, *P* < 0.05; **, *P* < 0.01, ***, *P* < 0.001. G represents Genotype; Si represents Silicon treatment.

**TABLE S6．** Two-way ANOVA of enzyme activities in rice roots as influenced by genotype and Si treatment

| Enzymes | Source | Time Points (h) | | | | | |
| --- | --- | --- | --- | --- | --- | --- | --- |
|  |  | 0 | 3 | 6 | 12 | 24 | 48 |
| CAT | G | 0.057 | 0.001** | 0.694 | 0.446 | < 0.001*** | 0.041* |
|  | Si | 0.705 | 0.280 | 0.137 | 0.681 | 0.004** | 0.911 |
|  | G × Si | 0.705 | 0.234 | 0.317 | 0.176 | 0.002** | 0.737 |
| SOD | G | 0.351 | 0.275 | 0.121 | 0.026* | 0.006** | 0.102 |
|  | Si | 0.194 | 0.260 | 0.051 | 0.435 | 0.074 | 0.590 |
|  | G × Si | 0.498 | 0.573 | 0.180 | 0.181 | 0.036* | 0.515 |
| POD | G | 0.054 | 0.026* | 0.033* | 0.066 | 0.005** | 0.122 |
|  | Si | 0.016 | 0.008** | 0.005** | 0.220 | 0.002** | 0.003** |
|  | G × Si | 0.911 | 0.307 | 0.385 | 0.002** | 0.057 | 0.157 |
| PPO | G | 0.138 | 0.076 | 0.017* | 0.019* | 0.009** | 0.002** |
|  | Si | 0.439 | 0.306 | 0.004** | 0.087 | 0.003** | 0.001** |
|  | G × Si | 0.377 | 0.358 | 0.079 | 0.400 | 0.338 | 0.104 |

*, *P* < 0.05; **, *P* < 0.01, ***, *P* < 0.001. G represents Genotype; Si represents Silicon treatment.
